# Neural stem cell-derived extracellular vesicles favour neuronal differentiation and plasticity under stress conditions

**DOI:** 10.3389/fnmol.2023.1146592

**Published:** 2023-03-24

**Authors:** Susana Delgado Ocaña, Dario Magaquian, Claudia Banchio

**Affiliations:** Laboratorio de Biología Molecular y Celular de Lípidos, Instituto de Biología Molecular y Celular de Rosario (IBR-CONICET) Ocampo y Esmeralda, Predio CONICET and Departamento de Ciencias Biológicas, Facultad de Ciencias Bioquímicas y Farmacéuticas, Universidad Nacional de Rosario, Rosario, Argentina

**Keywords:** extracellular vesicles, neural stem cell, neuronal differentiation, dendritic spines, neuronal plasticity, stress conditions

## Abstract

Extracellular vesicles (EVs) are released by all cell types and are involved in intercellular communication. We evaluated if neural stem cells-derived EVs (NSC-EVs) regulate NSCs proliferation and differentiation under control and stress conditions. We found that NSC-EVs treatment increases cell proliferation and promotes neuronal differentiation and plasticity. The fact that nervous tissue poorly recovers after cellular damage, prump us to evaluate the effect of EVs supplementation under oxidative stress and inflammation. We demonstrate that NSC-EVs restore the proliferative potential of the NSCs affected by oxidative stress. In addition, we provide evidence that oxidative stress and inflammation induce neuronal differentiation. Interestingly, the aberrant cell phenotype induced by inflammation is restored by NSC-EVs treatment, suggesting that these vesicles ameliorate the damage burden in neurons and modulate neuronal plasticity. These results contribute to understand the role of the NSCs-derived EVs as key players for brain tissue generation and regeneration and open new pathways to the development of therapies.

## 1. Introduction

Neural stem cells (NSC) have the potential of self-renewal or differentiation in all the main cell types of the nervous tissue including neurons, astrocytes, and oligodendrocytes (Han et al., 2016). This potential can be strongly influenced by extrinsic signals, including cytokines, growth factors and neurotrophins (Andreotti et al., 2019). Thus, after activating certain intrinsic pathways, the progenitor cells are driven towards a particular fate. The environment, meanwhile, can further refine the cell fate by inducing changes in gene expression (Watanabe and Raff, 1990; Desai and McConnell, 2000; Reik, 2007).

Neurological disorders and neurodegenerative diseases are caused by, or accompanied with oxidative stress and/or inflammation that causes a major and irreversible loss of neurons (Fischer and Maier, 2015). In this context, mounting evidence support that stem cell-based therapies could be promising on the basis of their potential for cell differentiation and replacement (Trounson and McDonald, 2015; Han et al., 2016). However, preclinical studies have suggested that very few injected cells (<1%) could survive or differentiate over 4 weeks after transplantation because of the hostile, injured microenvironment (Eggenhofer et al., 2012). In recent years, emerging evidences suggests that the secretome of NSCs is a promising alternative that might ensure more efficient outcomes than stem cell-based therapies (Cossetti et al., 2012). As constituents of the secretome, extracellular vesicles (EVs) are a key part of the NSC niche (Ma et al., 2019; Zhang et al., 2020) and mediate the intercellular communication (Mathieu et al., 2019). EVs carry various cell-derived bioactive molecules, including proteins, nucleic acids, lipids and metabolites (Johnstone, 2006), and deliver functional molecules to recipient cells resulting in the alteration of their control and pathological functions (Yokoi and Ochiya, 2021). Various *in vitro* and *in vivo* studies have demonstrated that the EVs derived from stem cells display the main features of parent stem cells holding a therapeutic potential (Lai et al., 2010; Xin et al., 2012).

Here, we demonstrated that EVs derived from NSCs (NSC-EVs) promote NSCs proliferation under control conditions and also rescue this capacity under oxidative stress. We also evaluated how EVs can influence the process of NSCs differentiation under control and two different stress conditions (oxidative stress and inflammation). We demonstrated that NSC-EVs promote neuronal differentiation without affecting astroglial differentiation. More importantly, EVs treatment restores the aberrant phenotype induced by inflammation by increasing morphological and functional parameters involved in neuronal maturation and synapses. In conclusion, our results suggest that NSC-EVs promote neural stem cell proliferation, differentiation and maturation under control and damage conditions. This finding provides novel data of the role of EVs and could encourage future development of free-cell therapies.

## 2. Materials and methods

### 2.1. Chemicals and antibodies

Dulbecco’s modified medium/Ham’s F12 (DMEM/F12 1:1), Dulbecco’s Modified Eagle’s Medium (DMEM), B27 and anti-rabbit Alexa Fluor® 488-labelled were purchased from Life Technologies Corporation (Carlsbad, CA, Unites States). Foetal bovine serum (FBS) from Internegocios (Buenos Aires, Argentina) and (DCFH-DA) from Molecular Probes. Hydrogen Peroxide was purchased from Cicarelli (Santa Fe, Argentina). Rabbit anti-βIII-tubulin antibody, protease inhibitor cocktail, poly-D-lysine (PDL), epidermal growth factor (EGF) and human basic fibroblast growth factor (bFGF) were purchased from Sigma (St. Louis, MO, United States). Mousse anti-synaptophysin, mousse anti-β-Actin, mouse anti-GFAP, mouse anti-ALIX, mouse anti-TSG101 and mouse anti-HSP70 from Santa Cruz Biotechnology (Dallas, Texas, United States); rabbit anti-PSD-95 and Vybrant™ DiI Cell-Labelling Solution from Invitrogen. Rabbit anti-Olig2 and mouse anti-Cy3-labelled from Millipore (Massachusetts, United States).

### 2.2. Isolation and culture of fetal neural stem cells

Neural stem cells were obtained from mouse fetal brain tissue as previously described (Costa et al., 2008). Briefly the lateral portion of the dorsal telencephalon (cortex) of embryonic day 13 (E13) C57/BL6 mice were isolated and chemically disrupted with trypsin (5 min, 0.05% w/v) and then mechanically disrupted into single cells by repeated pipetting in medium DMEM/F12 (1:1) containing 10% FBS, penicillin G (100 units/mL) and streptomycin (100 μg/mL). After centrifugation (5 min, 1,000 rpm) the pellet was resuspended in serum-free medium DMEM/F12 (1:1) and dissociated cells were cultured at a density of 5 × 10^4^ cells/mL in proliferation medium containing DMEM/F12 (1:1) supplemented with B27, 10 ng/mL bFGF and 10 ng/mL EGF and maintained at 37°C in a humidified 5% CO_2_ incubator. After 7 days, primary neurospheres were collected by low-speed centrifugation and dissociated chemically and mechanically into single cells. Then cells were re-plated to obtain a new passage: all the experiments were performed using cells from second passage. Neural stem cell differentiation was performed by plating 2.5 × 10^5^ or 5 × 10^4^ single cells on poly-D-lysine (PDL) (10 μg/mL)-coated 24 or 96 well plates, respectively, in differentiation medium containing DMEM/F12 (1:1) supplemented with B27.

All animal experiments and related protocols were approved by the Bioethics Commission for the Management and Use of Laboratory Animals of National University of Rosario, Argentina (N 6060/89). All procedures were carried out in accordance with the approved guidelines (Guide for the care and use of Laboratory Animals-8° edition-e National Academies Press-Washington DC 2011 and in compliance with the ARRIVE guidelines).

### 2.3. Extracellular vesicles isolation, purification and characterisation

NSC-EVs were purified using size exclusion chromatography qEVoriginal (Izon Science, Medford, MA, United States) according to manufacturer’s instructions. Briefly, NSCs supernatants were centrifuged at 500 g for 10 min and then 10,000 g for 10 min to remove cell debris and passed through a 0.22 μm filter to remove larger vesicles and cellular debris. Cleared conditioned media was concentrated to a final volume of 500–1,000 μl using AmiconR Ultra-15 100,000 kDa filters (Millipore Sigma). EVs separation was performed after column equilibration at room temperature. Fractions of 0.5 mL were collected using PBS as eluent. Four EV-rich fractions (8–11) were pooled and either analysed directly or concentrated using an AmiconR Ultra-4,100 kDa centrifugal filters.

Negative staining of exosome suspensions followed by imaging in a transmission electron microscope (TEM) was used to determine vesicle shape and size distribution. Briefly, 10 μl aliquots of EVs suspension were dispensed onto sheets of Parafilm in a humidified petri dish and the vesicles were deposited on carbon-coated grid (300–400 mesh) for 3–5 min. Afterwards, the sample was negatively stained with 2% uranyl acetate for 3–5 min. The excess stain was removed with filter paper and the grid was briefly washed with Mili-Q water and air-dried for 15 min at RT. Images were taken using a Zeiss EM 109 T accoupled to digital camera Gatan ES 1000 W, at the Facility for Transmission Electron Microscopy of the LANAIS-MIE (UBA-CONICET).

For EVs quantification, EVs were lysed with Tris-SDS Buffer (2% sodium dodecyl sulphate, 20 mM Tris–HCL pH 8) and heated at 95°C for 5 min, and proteins content was measured using BCA protein assay kit (Thermo Fisher, United States). Exosome-Protein markers HSP 70 (1/500), Alix (1/500) and TSG 101 (1/500) were determined by immunoblotting. In addition, dynamic light scattering (DLS) was performed to determinate EVs size.

### 2.4. Macrophages culture and LPS-induced stimulation

The mouse cell line Raw 264.7 (ATCC® TIB-71™) was cultured in DMEM 10% FBS supplemented with penicillin G (100 units/mL), streptomycin (100 μg/mL) (proliferation conditions) and maintained in a 5% CO2 humidified incubator at 37°C. Activation with lipopolysaccharide (LPS) was performed according was previously described (Magaquian et al., 2021). Briefly, cells were grown to 80% confluence in petri dishes with DMEM medium supplemented with 10% FBS. After centrifugation at 1500 rpm for 10 min the cell pellet was resuspended in 1 mL of DMEM/F12 medium and cells were transferred to a new plate containing DMEM/F12 stem cell medium (in the absence of FBS, B-27 and growth factors). Pure LPS was added in a final concentration of 1 μg/mL. After 18 h of incubation, cells were centrifuged at 1000 rpm for 5 min and the culture medium was filtered through 0.22 μm filters (Sartorius) and stored at − 80°C until use.

### 2.5. Cell viability and proliferation assays

Cell viability was assessed by MTT-reduction assay (Mosmann, 1983). After cell treatment, MTT (5 mg/mL) was added to the cell culture medium at a final concentration of 0.5 mg/ml and incubated for 4 h at 37°C, 5% CO_2_. The assay was stopped by replacing the MTT-containing medium with DMSO. The extent of MTT reduction was measured spectrophotometrically at 570 nm. Results are expressed as a percentage of the control.

Proliferation of NSCs was assayed by measuring the neurosphere’s diameter (Rietze and Reynolds, 2006). Briefly, 5,000 living cells were seeded per well in 24-well plates and cultured for up to 96 h to evaluate the expansion rates. Size of 100 neurospheres (expressed as neurospheres diameters) was measured in three independent experiments. Images were taken with a microscope Olympus BH-2 and analysed using the freeware image J (National Institutes of Health, freeware).

### 2.6. Immunocytochemistry

Cells were cultured on PDL (10 μg/mL)-coated glass coverslips in 24-well plates as previously described (Montaner et al., 2018). After the desired incubation period, cells were fixed in 4% (w/v) paraformaldehyde-sucrose for 30 min at room temperature, permeabilized with 0.2% Triton X100 and blocked for 1 h in 5% BSA. Cells were incubated with the primary antibody overnight at 4°C followed by incubation with the fluorescently labelled secondary antibody for 1 h at room temperature. Primary and secondary antibodies were diluted as follows: rabbit anti-β-III tubulin (1:500), mouse anti-GFAP (1:250), rabbit anti-Olig 2 (1:500), mouse anti-Synaptophysin (1:300), anti-rabbit Alexa Fluor_®_ 488-labelled (1:500) and anti-mouse Cy3-labelled (1:300). To visualize nuclei, cells were counterstained and mounted with ProLong Gold antifade reagent containing DAPI (Molecular probes, Life technologies).

### 2.7. Microscopy and image analysis

Micrographs were acquired using a confocal microscope (Zeiss LSM 880) or the Nikon Model Eclipse 800 microscope and quantitative analyses were performed with Image J (NIH). Cells were counted from 20 randomly selected fields per well for each individual experiment. At least three independent experiments were performed. The percentage of neuronal cell population was calculated against the DAPI-positive total cell number which includes undifferentiated stem cells and differentiated neurons. Cells bearing at least one neurite equal or longer than the soma diameter were considered to be differentiated. The number of synaptophysin containing vesicles and neurite lengths was measured and counted manually. To differentiate dystrophic and normal neuronal populations,100 neurons from each experiment were manually selected and analysed.

### 2.8. Western blot analysis

For Western blot experiment Neurosphere-derived cells were plated at a density of 1.5 10^5^ cell and cultured on PDL-coated 35 mm culture dishes under differentiation conditions. After 72 h, cells were collected, resuspended in lysis buffer (50 mM Tris–HCl pH 8.0, 50 mM KCl, 10 mM EDTA, Nonidet P-40 1%, 20 mM NaF, 1 mM Na_3_VO_4_, 1 mM PMSF and 1:1000 protease inhibitor cocktail) and sonicated five times at 5% amplitude for 5 s (Sonics and Materials Inc–Vibra CellTM). Protein concentration was determined using bovine serum albumin (BSA) as standard protein and “PierceTM BCA Protein Assay Kit (Thermo Scientific).” 20 μg of cell lysate were resolved on 12% SDS-polyacrilamide gel electrophoresis (PAGE) and transferred to a nitrocellulose membrane (Amersham, GE Healthcare). After blocking overnight with 5% non-fat milk in 0.1% Tween TBS and washing, blots were incubated with anti-synaptophysin (1:300), anti-PSD95 (1/300), anti-overnight at 4°C. Peroxidase-conjugated anti-mouse IgG (1:8000, Jackson Immuno Research) was used as secondary antibody. For loading protein control anti-β-Actin (1:6000) was used and developed with secondary antibody peroxidase-conjugated anti-mouse IgG (1,8,000, Jackson Immuno Research). Labelled proteins were detected with chemiluminescence reagents (AmershamTM ECLTM Prime Western Blotting Detection Reagent, GE Healthcare).

### 2.9. Labelling of EVs

For labelling, 25 ul of EVs in PBS were mixed with Vybrant™ DiI Cell-Labelling Solution (Thermo Fisher) (1:9) and incubated at room temperature for 30 min. Next, Dil-EVs were added to NSC, and the uptake of EVs was immediately monitored by *in vivo* confocal microscopy (Zeiss LSM 880) and qualitative analyses were performed with Image J” (NIH).

### 2.10. Qualitative evaluation of intracellular ROS levels

To evidence the presence of intracellular reactive oxygen species (ROS), the fluorescent probe 2′,7′-dichlorodihydrofluorescein diacetate (DCFH-DA) was used. To this end, 250,000 NSCs seeded in 24-well plates under differentiation conditions were supplemented with 50 μM of H_2_O_2_ for 30 min, washed and incubated during 60 min in the presence or in the absence of EVs (7.5 μg of NSC-EVs-containing proteins). After three washes with PBS, cells were incubated with 80 μM DCFH-DA probe in the dark for 30 min. After this time, cells were washed the cells with PBS three times and fluorescence was observed using the Nikon Eclipse 800 fluorescence microscope (ʎe = 480 nm).

### 2.11. Statistical analysis

Data represent the mean value ± SEM of at least three independent experiments and each individual experiment was performed in technical triplicate. Statistical significance was determined by either Student’s t-test or One-Way ANOVA followed by Tukey’s test using Prism (GraphPad Software Inc.,). *p*-values lower than 0.05 were considered statistically significant.

## 3. Results

### 3.1. Extracellular vesicles purification and characterization

**Figure 1:**
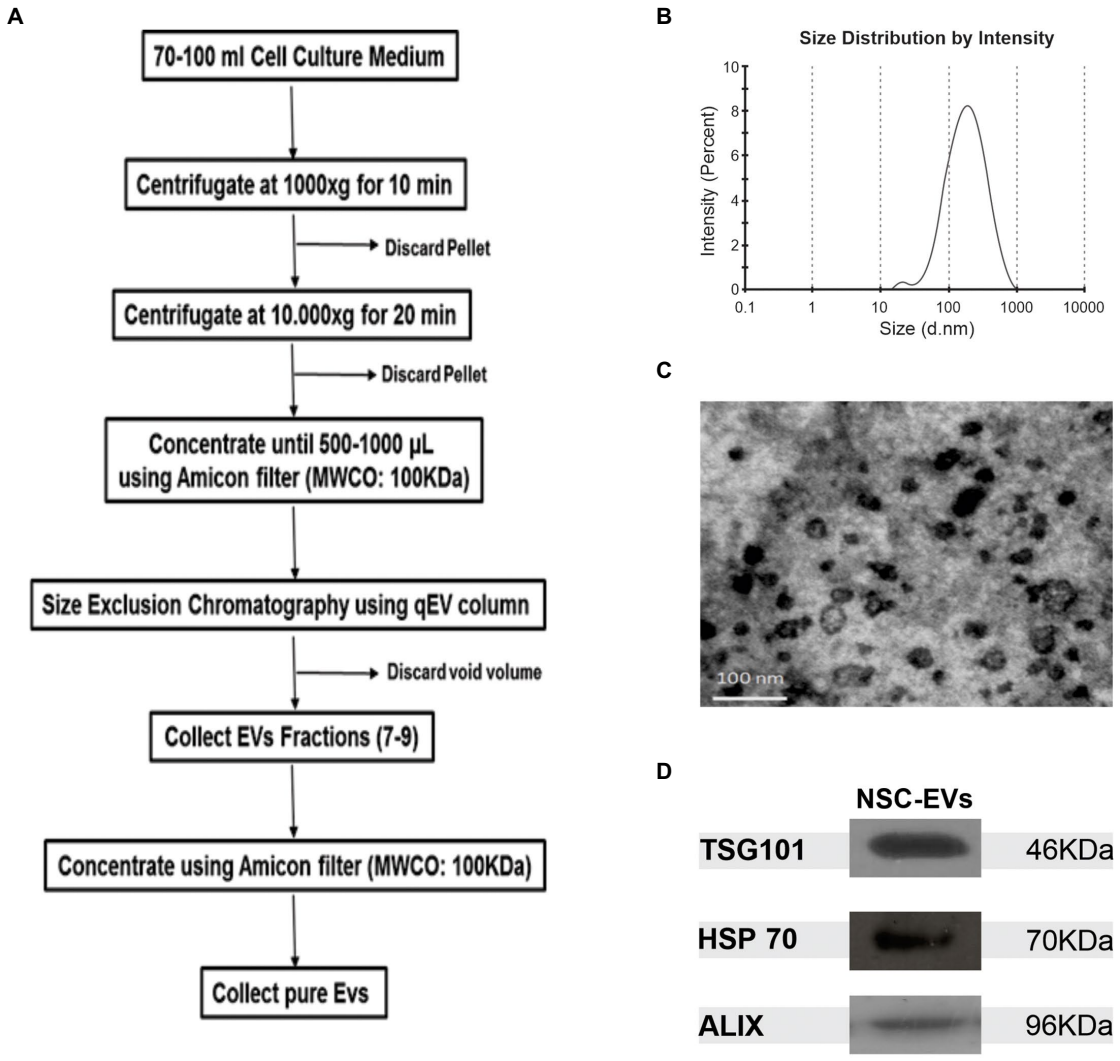
NSCs-EV purification and characterisation. **(A)** Protocol to purify NSC-EVs by sequential centrifugation and size exclusion chromatography. **(B)** Size distribution of NSC-EVs measured by DLS. **(C)** Representative image of electron micrograph of purified NSC-EVs. **(D)** Representative image of Western Blot analysis of purified NSC-EVs. The EVs markers TSG101, HSP70 and ALIX were detected.

To purify high quality EVs from NSCs cultured under proliferation, we used size exclusion chromatography (qEVoriginal IZON Science) according to manufacturer’s instructions (Figure 1A). Dynamic light scattering (DLS) revealed that 97,2% of the purified EVs have an average size of 164,2 nm (Figure 1B), and transmission electron microscopy (TEM) analysis showed their donut-shaped morphology (Figure 1C). We also searched for the presence of vesicular/exosomal proteins like Alix, TSG101 and HSP70 (Witwer et al., 2013) by Western Blot (Figure 1D). Taken together, the foregoing data suggests that the method allows the efficient and straight-forward isolation of EVs that are enriched in exosomes, but we simply refer to them as EVs. In addition, all these analyses confirmed that they are suitable for use in the subsequent assays and experiments.

### 3.2. Effect of NSCs extracellular vesicles in NSCs proliferation under control and different stress conditions

**Figure 2:**
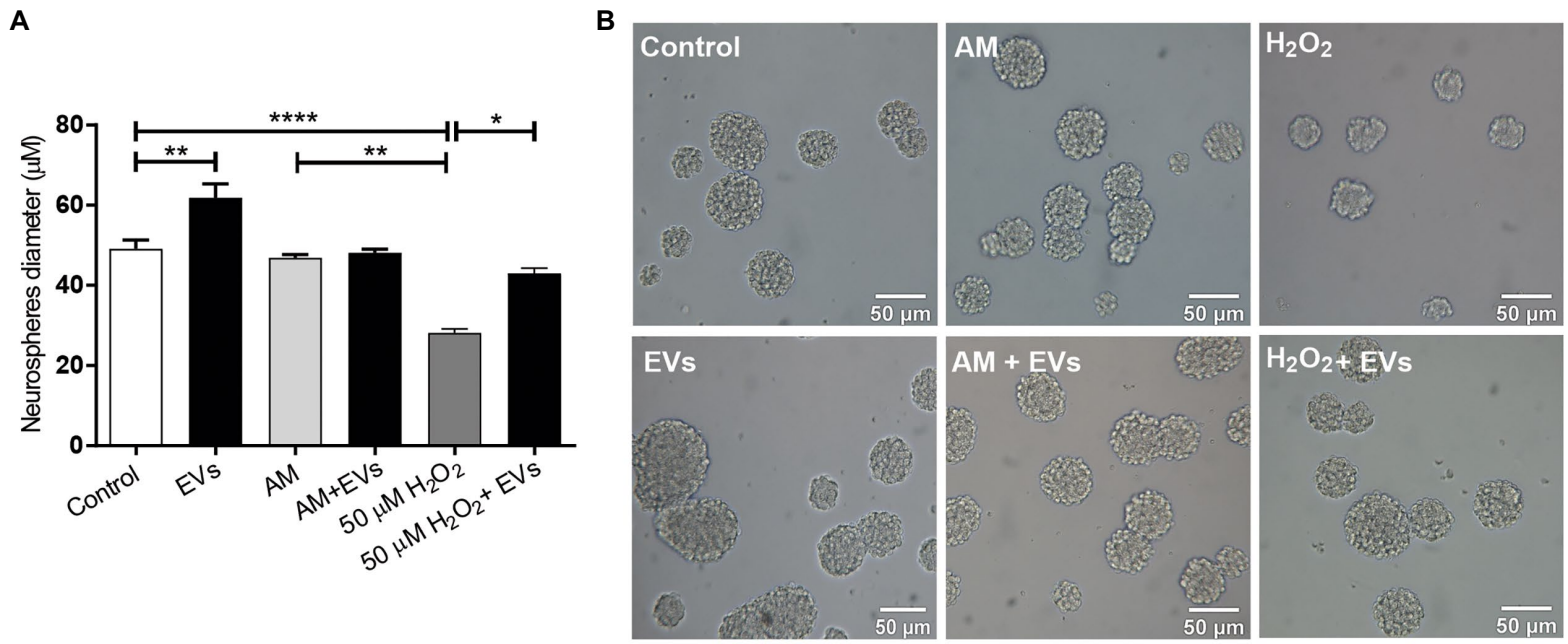
NSC-EVs regulate NSCs proliferation. **(A)** After incubating the NSCs under the indicated conditions (H_2_O_2_ 50 μM; AM: activated inflammatory media) in the presence or in the absence of extracellular vesicles (NSC-EVs) during 96 h, proliferation was analysed by measuring neurosphere’s diameter. Graph represents the neurosphere’s diameter measured in three independent experiments. Data were presented as mean ± SEM. ^****^p < 0.0001; ^**^p < 0.01; ^*^p < 0.05. **(B)** Representative images (20X) of neurospheres incubated in the indicated conditions. Scale bars: 50 μm.

Proliferation and renewal of NSCs is key for tissue regeneration (Ottoboni et al., 2020). We analysed if NSC-EVs could act as an autologous communication signal regulating its own proliferation rate. We first analysed if NSCs internalided NSC-EVs. To that end, NSC-EVs were labelled with Vybrant™ DiI Cell-Labelling Solution (Thermo Fisher) (1:9 v/v) and their fate was evaluated by time lapse microscopy measuring red fluorescence using the confocal microscope (Zeiss LSM 880); the qualitative analysis was performed with Image J” (NIH) (Supplementary video). After confirming that NSC-EVs target NSCs, we evaluated cell proliferation by incubating NSCs under proliferation conditions (neurosphere culture) in the presence or in the absence of NSC-EVs (7.5 μg of NSC-EVs-containing proteins). After 96 h, we measured the neurosphere’s diameter as a parameter of cell proliferation (Magaquian et al., 2021). As shown in Figure 2, there is a clear and significant increase in the neurosphere’s diameter promoted by NSC-EVs.

Reactive oxygen species (ROS) like H_2_O_2_ are released in response to neural damage (Uttara et al., 2009). To determine the concentration of peroxide that caused moderate oxidative damage, we incubated NSCs in the presence of different concentrations of H_2_O_2_ during 30 min and evaluated cell viability by MTT (Mosmann, 1983) (Supplementary Figure S1). We defined 50 μM of H_2_O_2_ during 30 min as a concentration that causes a moderate damage to the neurospheres and confirmed using DCFH-DA as a probe, that under the selected condition there is an increase in the intracellular ROS (Aranda et al., 2013) (Supplementary Figure S1). We incubated NSCs with H_2_O_2_ and demonstrated that this ROS specie negatively affects NSCs proliferation inducing a significant decrease in neurosphere diameter (Figure 2). Next, we evaluated if NSC-EVs could attenuate this process by performing the same experiment but after 30 min of H_2_O_2_ (50 μM) treatment, cells were incubated with fresh media containing NSC-EVs (7.5 μg of NSC-EVs-containing proteins). As Figure 2 shows, NSC-EVs restore the capacity of NSCs to proliferate under oxidative stress, probably by decreasing ROS accumulation (Supplementary Figure S1). Another characteristic of many neurodegenerative conditions is inflammation. We have previously demonstrated that NSCs-proliferation is not affected by ILs and INFα (activated media: AM) (Magaquian et al., 2021). Here we evaluate the effect of NSC-EVs and demonstrate no changes of proliferation under this specific condition (Figure 2).

### 3.3. NSC-EVs induces NSCs differentiation towards neurons

**Figure 3:**
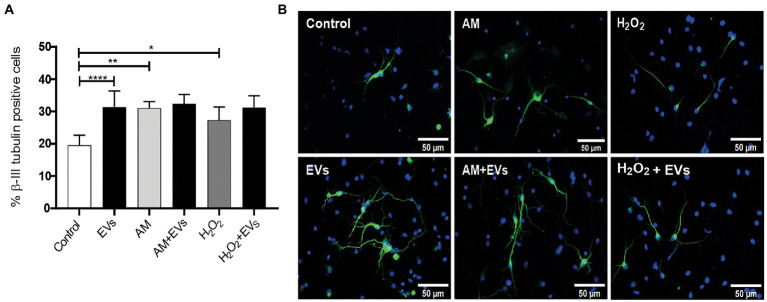
NSC-EVs induce neuronal differentiation. Neurosphere-derived cells were cultured during 3 days under differentiation conditions and immunostained with anti-βIII-tubulin antibody (green). Nuclei were counterstained with DAPI (blue). **(A)** Percentage of βIII-tubulin positive cells of NSCs exposed to oxidative stress (50 μM H_2_O_2_ or to 20% v/v of AM) in the presence or in the absence of EVs during 3 days were analysed by immunocytochemistry coupled to fluorescence microscopy. Graph represents the percentage of neuronal differentiation measured in five independent experiments. Data were presented as mean ± SEM. ^****^p < 0.0001; ^**^p < 0.01; ^*^p < 0.05. **(B)** Representative images (40X) of the immunofluorescence assays with the neuronal marker (βIII-tubulin, green) nuclei (DAPI, blue). Scale bars: 50 μm.

**Figure 4:**
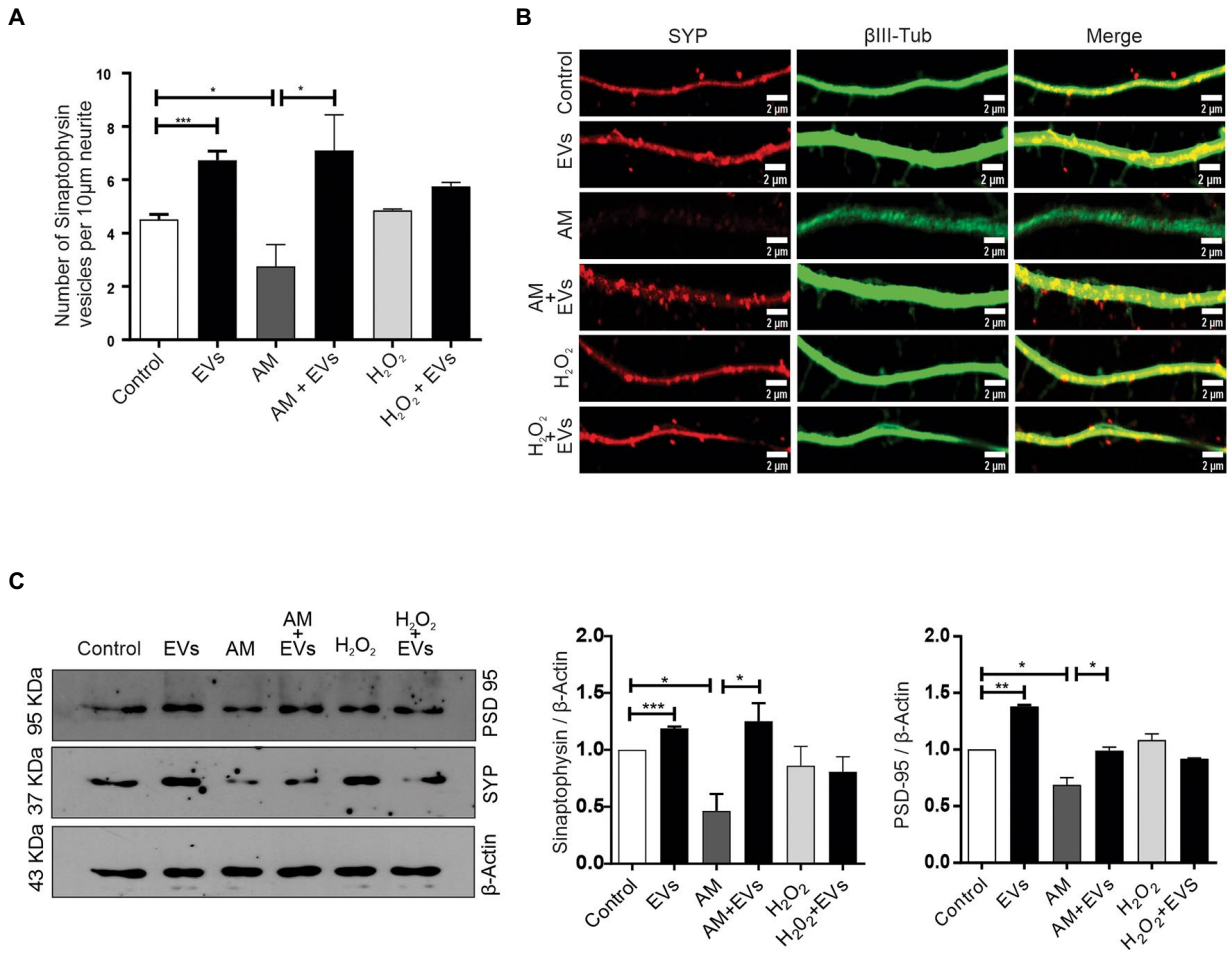
NSC-EVs promotes the expression of synaptic proteins. **(A)** Quantification of Synaptophysin vesicles. Graph represents the number of Synaptophysin-containing vesicles in 10 μm length measured in three independent experiments. Data were presented as mean ± SEM. ^**^p < 0.01; ^*^p < 0.05. **(B)** Representative images (63X) of the immunofluorescence assays with Synaptophysin (red) and the neuronal marker (βIII-tubulin, green) taken after 3 days of incubation in the indicated conditions. Scale bars: 1 μm. **(C)** Representative image of Western Blot showing Synaptophysin (SYP) and PSD-95 levels in NSCs exposed under the indicated condition during 72 h. β-actin was used as control loading. The gels/blots displayed here are cropped, and without high-contrast (overexposure). Densitometry analysis was performed and the statistical differences were evaluated using Student’s T-test. ^***^p < 0.001; ^**^p < 0.01; ^*^p < 0.05.

**Figure 5:**
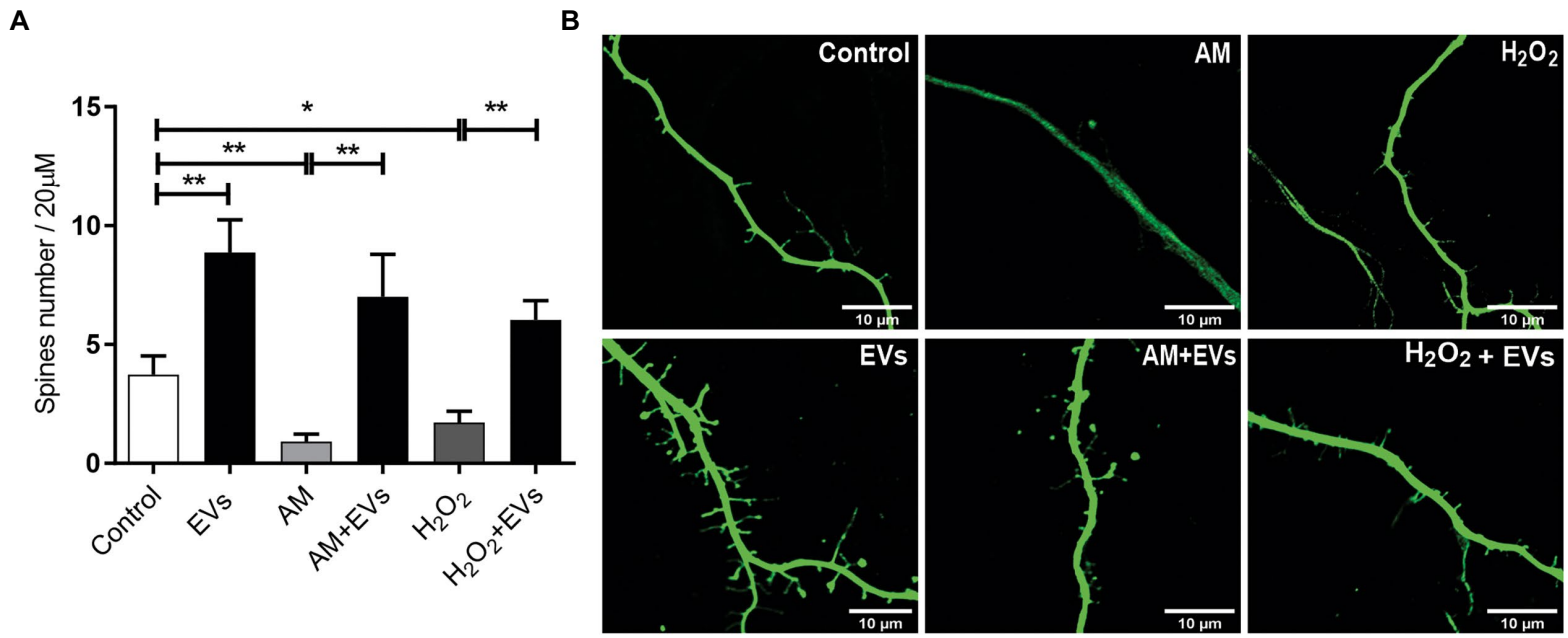
NSC-EVs promotes dendritic spines development even under stress conditions. **(A)** Quantification of spine number *per* 20 μm of neurite length. Values of quantification are expressed as mean ± SEM ^*^p < 0.05, ^**^p ≤ 0.01. **(B)** Representative images of dendritic spines (63X) under the indicated conditions after 3 days. Scale bar: 10 μm.

The balance between neuronal and astroglial differentiation plays a relevant role during tissue generation and regeneration (Xiao et al., 2014). To evaluate the role of NSC-EVs under control condition, we analysed neuronal and glial differentiation of NSCs incubated with NSC-EVs. We performed immunocytochemistry followed by microscopic analysis of the percentage of cells that express βIII-tubulin as neuronal lineage marker, GFAP as astrocyte marker and Olig-2 as oligodendrocyte marker (Montaner et al., 2018). We observed that after 72 h of NSC-EVs (7.5 μg of NSC-EVs-containing proteins) treatment promotes neuronal differentiation (Figure 3) but do not affect neither astrocyte nor oligodendrocyte differentiation. However, EVs isolated from Neuro-2a and HEK-293 do not affect neuronal differentiation suggesting that this effect is a characteristic of NSC-EVs (Supplementary Figure S2). In addition, a detailed morphometric analysis revealed that NSC-EVs treatment increases the average of the main neurite lengths and total neurites length (Supplementary Figure S3). In addition, NSC-EVs impact in parameters associated with neuronal function; we demonstrated an increase in the number of Synaptophysin-containing vesicles (Figure 4A), and in the level of expression of Synaptophysin and the post-synaptic protein PSD-95 (Figures 4B,C). Furthermore, we quantified the number of dendritic spines and demonstrate a significant increase when cells were incubated with NSC-EVs (Figure 5).

### 3.4. NSC-EVs restore neuronal differentiation In stress

**Figure 6:**
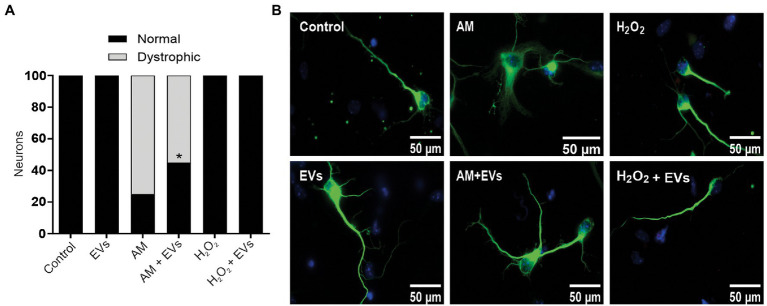
NSC-EVs restores morphology of dystrophic neurons. **(A)** Percentage of normal neurons (black bars) and dystrophic neurons (grey bars) after 3 days in culture medium under the indicated conditions. (Student’s T-test) ^*^p < 0.05. **(B)** Representative images of neurons incubated under the indicated conditions (100X) Scale bars: 50 μm.

The burden of multiple conditions that give rise to acute or chronic inflammation is neuronal death (Aktas et al., 2007). Considering our results showing that NSC-EVs treatment improves neuronal differentiation and induces favourable morphological and functional changes under conditions that mimic a physiological state, we speculated that NSC-EVs could have a beneficial impact on neuronal differentiation under stress. We have previously demonstrated that incubation of NSCs during 72 h in AM affects neuronal differentiation, morphology and function. In fact, under inflammation, there is a clear increase in NSCs differentiation towards neuronal lineage, but the resulting neurons are aberrant (Magaquian et al., 2021). To evaluate the role of NSC-EVs under inflammation, we incubated NSCs with AM (20% V/V) (Magaquian et al., 2021) in the presence and in the absence of NSC-EVs. The analysis of βIII-tubulin expressing cells indicates that NSC-EVs treatment does not further increase the percentage of neurons but restores the aberrant phenotype observed under inflammation (Figures 3A,B, 6). Furthermore, a deeper analysis of functional parameters shows that NSC-EVs restore the number of Synaptophysin containing vesicles (Figures 4A,B), the levels of Synaptophysin and PDS-95 expression (Figure 4C) and the number of dendritic spines (Figure 5), suggesting that NSC-EVs could be used as a tool to stimulate tissue regeneration and neuronal plasticity. Similarly, we evaluated the effect of ROS species on differentiation and demonstrated that H_2_O_2_ induces the rate of neuronal differentiation (Figure 3A). Even though the morphology of the neurons look similar to that observed in the control (see Figures 3B, 6), a detailed analysis demonstrated that those neurons have reduced neurite length and less dendritic spines than the control, and that treatment with NSC-EVs restores the levels of both parameters (Figure 5; Supplementary Figure S3).

## 4. Discussion

We showed that NSCs release EVs and that our protocol allows efficient and rapid purification of them. The purified NSC-EVs are enriched in exosomes as evidenced by the presence of exosome markers (TSG101, Alix and HSP70), and the expected shape and size range (Figure 1). We proved that NSC-EVs are taken up by NSCs, and that this autocrine intercellular communication modified the NSCs proliferation and differentiation potential, thus demonstrating that EVs derived from NSCs hold therapeutic potential. NSC-EVs treatment of NSCs incubated under proliferation conditions induces cellular proliferation (Figure 1). As the reservoir of NSCs is essential for tissue regeneration, perhaps this mechanism might contribute to maintain the NSCs pool under physiological conditions (Ottoboni et al., 2020).

Oxidative stress-induced apoptosis in NSCs has often been observed during various senile diseases (Lin et al., 2004; Harman, 2006; Saxena et al., 2019). In fact, we show that ROS accumulation negatively affects cell proliferation and survival (Figure 1; Supplementary Figure S1) and could be one of the reasons for the poor outcome of stem cell-based therapies (Trounson and McDonald, 2015; Han et al., 2016). Interestingly, treatment with NSC-EVs restores the rate of proliferation to control levels, showing that NSC-EVs treatment could have beneficial effects, specially under oxidative damage (Figure 2). A preventive effect on oxidative stress-induced senescence was also demonstrated using mesenchymal stem cell-derived exosomes (Matsuoka et al., 2021).

As previously described, inflammation has no effect on cell proliferation (Magaquian et al., 2021) and under this stress condition, NSC-EVs have no effect on cell proliferation as was observed under control conditions (Figure 2). Further analysis needs to be done to answer why, but we hypothesise that under inflammation, any effector component of EVs that promote cell proliferation could be negatively affected.

In our experiments, and by the first time to the best of our knowledge, we demonstrated that NSC-EVs treatment increases NSCs differentiation towards the neuronal lineage (Figure 3A) without affecting astrocyte and oligodendrocyte differentiation (Supplementary Figure S2). In addition, NSC-EVs increase the average length of neurites (Supplementary Figure S3), the levels of Synaptophysin and PSD-95 expression (Figure 4) and the density of dendritic spines (Figure 5). This important role of EVs has also been involved in axonal pathfinding (Gong et al., 2016) and synaptic pruning (Bahrini et al., 2015). Considering this favourable effect of NSC-EVs under control conditions, we asked whether these outcomes could also be effective under stress conditions and thus, favour tissue repair. We evaluated neuronal differentiation under inflammation and oxidative stress and determined that these conditions already increase the percentage of βIII-tubulin expressing cells (Figure 3A) and that NSC-EVs do not improve those percentages. As similar results were previously described (Pérez Estrada et al., 2014), this could be an endogenous response for neuronal regeneration. However, under inflammation, as was previously demonstrated (Magaquian et al., 2021), neurons show an aberrant phenotype (Figures 5, 6). Interestingly, even though NSC-EVs did not further increase the percentage of neurons, they clearly restore the morphology of aberrant neurons (Figures 5, 6). More importantly, NSC-EVs restore parameters associated with neuron function like Synaptophysin-containing vesicles, the levels of expression of Synaptophysin and PSD-95 (Figure 4) and the density of dendritic spines affected by stress conditions (Figure 5). The effect of NSC-EVs treatment on neuronal morphology and in the parameters associated with function described in the current manuscript, could explain the effectiveness of mesenchymal-EVs treatment ameliorating the damaged caused by traumatic brain injury (Ni et al., 2019; Williams et al., 2019). Different to our results, Ni et al. proposed that human neural-EVs favours recovery after stroke due to its anti-inflammatory effect (Ni et al., 2019).

In summary, our study demonstrates the effect of NSC-EVs on neural proliferation and differentiation under control and pathological conditions, and thus the importance of the microenvironment in nervous tissue generation and regeneration. Further studies will allow us to identify effector molecules carried by the NSC-EVs. Still, the information provided is shedding light on the development of NSC-EV-based therapeutic strategies to expand and activate neurogenesis *in vivo*, and very remarkable, to restore neuronal plasticity after inflammatory damage.

## Data availability statement

The raw data supporting the conclusions of this article will be made available by the authors, without undue reservation.

## Ethics statement

The animal study was reviewed and approved by CICUAL Facultad de Ciencias Bioquímicas y Farmacéuticas, UNR, Rosario, Argentina.

## Author contributions

SO and DM performed all the experiments and analysed data. CB designed and supervised research, acquired funding, and wrote the manuscript. All authors contributed to the article and approved the submitted version.

## Funding

This work was supported by Consejo Nacional de Investigaciones Científicas y Técnicas (CONICET), Agencia Nacional de Promoción Científica y Tecnológica (ANPCyT) (PICT 2017–0167 and PICT 2019–01073).

## Conflict of interest

The authors declare that the research was conducted in the absence of any commercial or financial relationships that could be construed as a potential conflict of interest.

## Publisher’s note

All claims expressed in this article are solely those of the authors and do not necessarily represent those of their affiliated organizations, or those of the publisher, the editors and the reviewers. Any product that may be evaluated in this article, or claim that may be made by its manufacturer, is not guaranteed or endorsed by the publisher.
