# Supplementary material for: Neural stem cell-derived extracellular vesicles favour neuronal differentiation and plasticity under stress conditions
Source: Front Mol Neurosci. 2023 Mar 24;16:1146592. doi: 10.3389/fnmol.2023.1146592 (PMC10080063; doi:10.3389/fnmol.2023.1146592)
Supplement: Supplementary file 1 [file Data_Sheet_1.docx]

**Supplementary Material**

**Figure 1:**

**B**

**A**


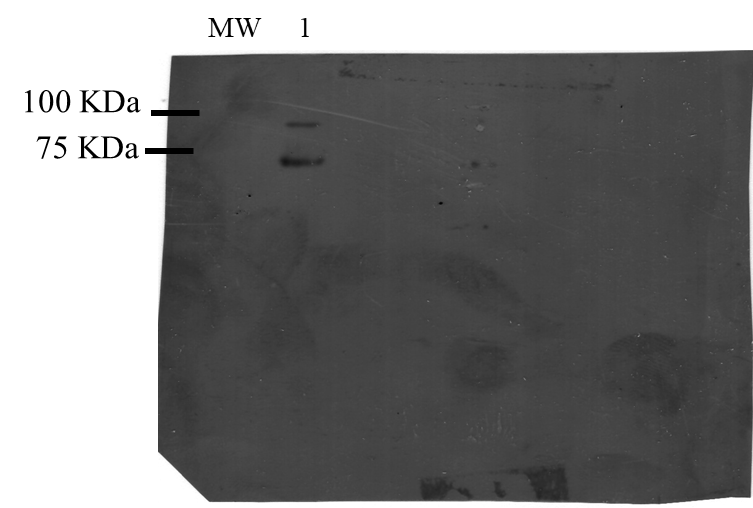

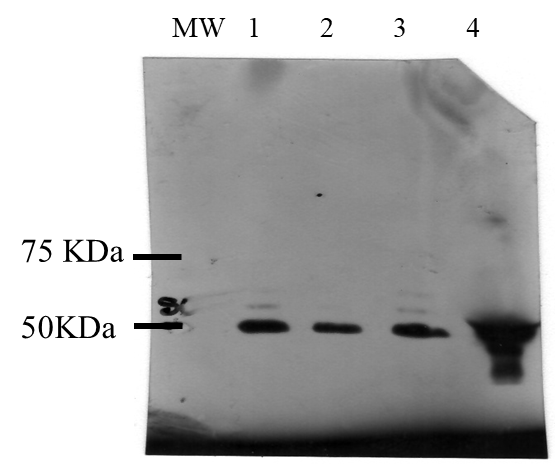


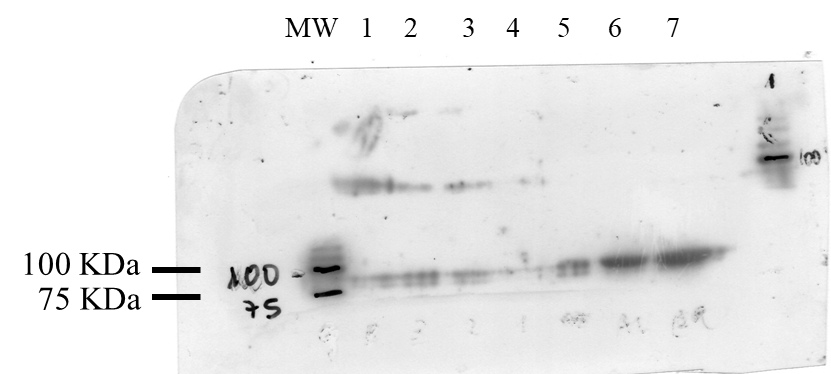


**C**

**Figure 1:** **NSCs-EV purification and characterization.** Western blots of purified NSC-EVs against exosomes markers. **(A)** TSG101 in 4 different preparations of EVs, MW: Molecular weight marker, 1-3: Extracellular vesicles purified using IZON column (See Materials and methods) and 4 using ultracentrifugation protocols. **(B)** HSP-70, MW: Molecular weight marker, 1: NSC-EVs purified using IZON columns. **(C)** ALIX. MW: Molecular wight marker, 1-5 fractions containing EVs purified using IZON columns (See Materials and methods). 6-7: Fractions containing EVs were concentrated by ultracentrifugation. For each gel, we loaded 10 µg of proteins in a 12 % SDS PAGE, transfer to nitrocellulose membrane (1.5 h/300mA) and blocked 1h in T-TBS milk 5%. Primary antibodies were incubated overnight at 4 °C followed by HRP- mouse secondary antibody (1/10000) during 1h. For detection, Amersham ECL Prime Western Blotting Detection Reagent and Rx Amersham Hyperfilm ECL were used.

**Figure 4:**

**A**

**
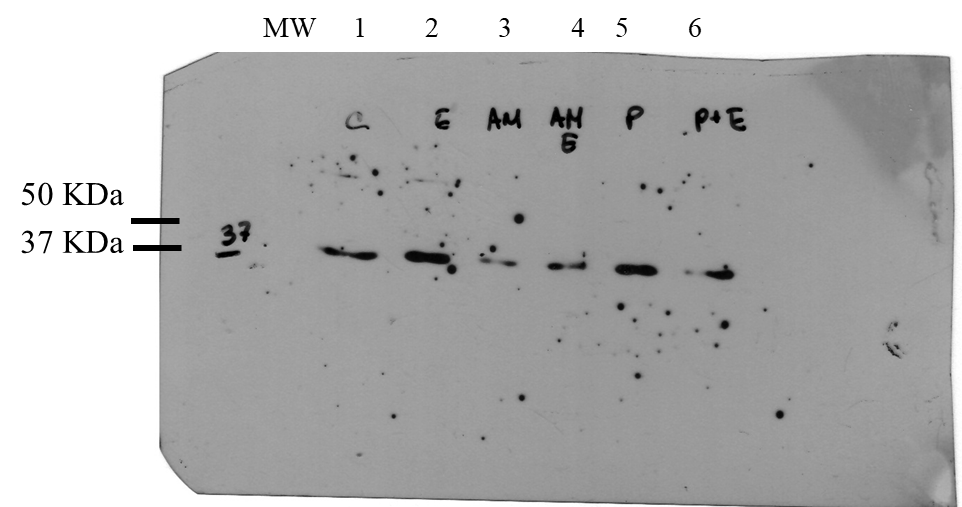
**

**B**

**
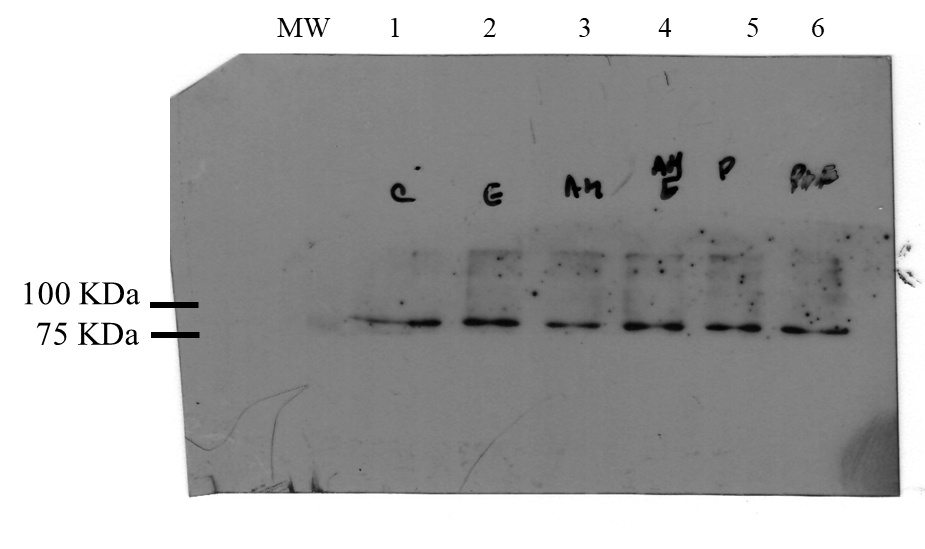
**

**C**

**
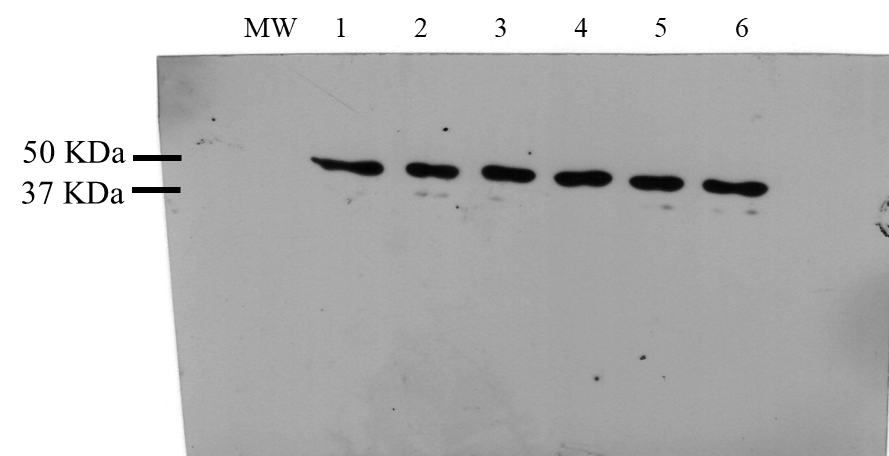
**

**Figure 4**: **NSC-EVs promotes the expression of synaptic proteins.**

Western blots showing **(A)** Synaptophysin and **(B)** PSD-95 levels of expression in NSCs treated or not with NSC-EVs under the indicated stress conditions. **(C)** β-Actin levels were measured as a control. 1: Molecular weight marker, 2: Control, 3: EVs, 4: AM, 5: AM+EVs, 6: H_2_O_2_, 7: H2O2+EVs. Briefly, we loaded 20 ug of proteins in a 12 % SDS PAGE, transfer to nitrocellulose membrane (1.5 h/300mA) and blocked 1h in T-TBS milk 5%. Membranes were incubated overnight at 4 °C with primary antibodies above-mentioned followed by HRP- mouse secondary antibody during 1h. For detection, Amersham ECL Prime Western Blotting Detection Reagent and Rx Amersham Hyperfilm ECL were used.
