# Supplementary material for: Neural stem cell-derived extracellular vesicles favour neuronal differentiation and plasticity under stress conditions
Source: Front Mol Neurosci. 2023 Mar 24;16:1146592. doi: 10.3389/fnmol.2023.1146592 (PMC10080063; doi:10.3389/fnmol.2023.1146592)
Supplement: Supplementary file 2 [file Data_Sheet_2.docx]

Supplementary Material

Neural Stem Cell-derived extracellular vesicles favour neuronal

differentiation and plasticity under stress conditions

Susana Delgado Ocaña, Dario Magaquian, Claudia Banchio*

*** Correspondence:** Claudia Banchio: banchio@ibr-conicet.gov.ar

**Supplementary Figures**:


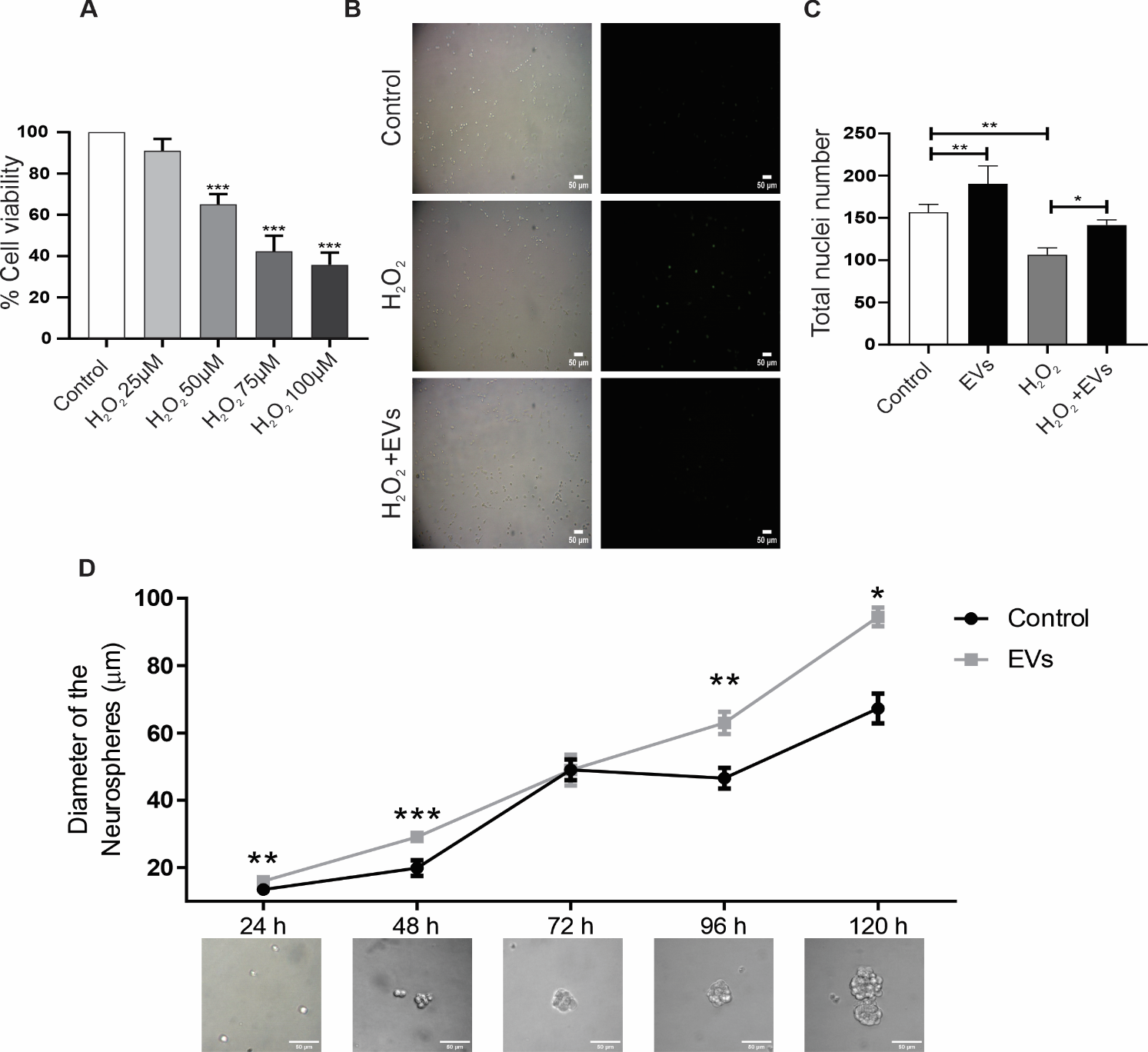


**Supplementary Figure 1: Characterization of oxidative stress damage**. **(A)** Graph represent the % of Cell viability analysed by MTT. Cells were incubated with increasing concentrations of Hydrogen peroxide (H_2_O_2_) during 30 minutes. After this time, cell culture media was changed and cells were incubated for 72 hours before analysis. **(B)** Representative images of NSCs stained with 50 µM of DCFH-DA to develop ROS species production. NSCs were supplemented with 50 µM of H_2_O_2_ for 30 minutes, and treated with NSC-EVs when indicated. (**C**) Number of cell nuclei. Cells were incubated with H_2_O_2_ (50 μM) and analysed by immunocytochemistry, the number of nuclei were quantified to confirmed the moderate damage induce by this stress condition and the recovery caused by EVs treatment. **(D)** Time course analysis of neurosphere’s diameter in the presence or in the absence of NSC-EVs. Data are presented as mean ± SEM of three independent experiments. Scale bars: 50 μm. *p < 0.05 **p < 0.01 ***p < 0.001


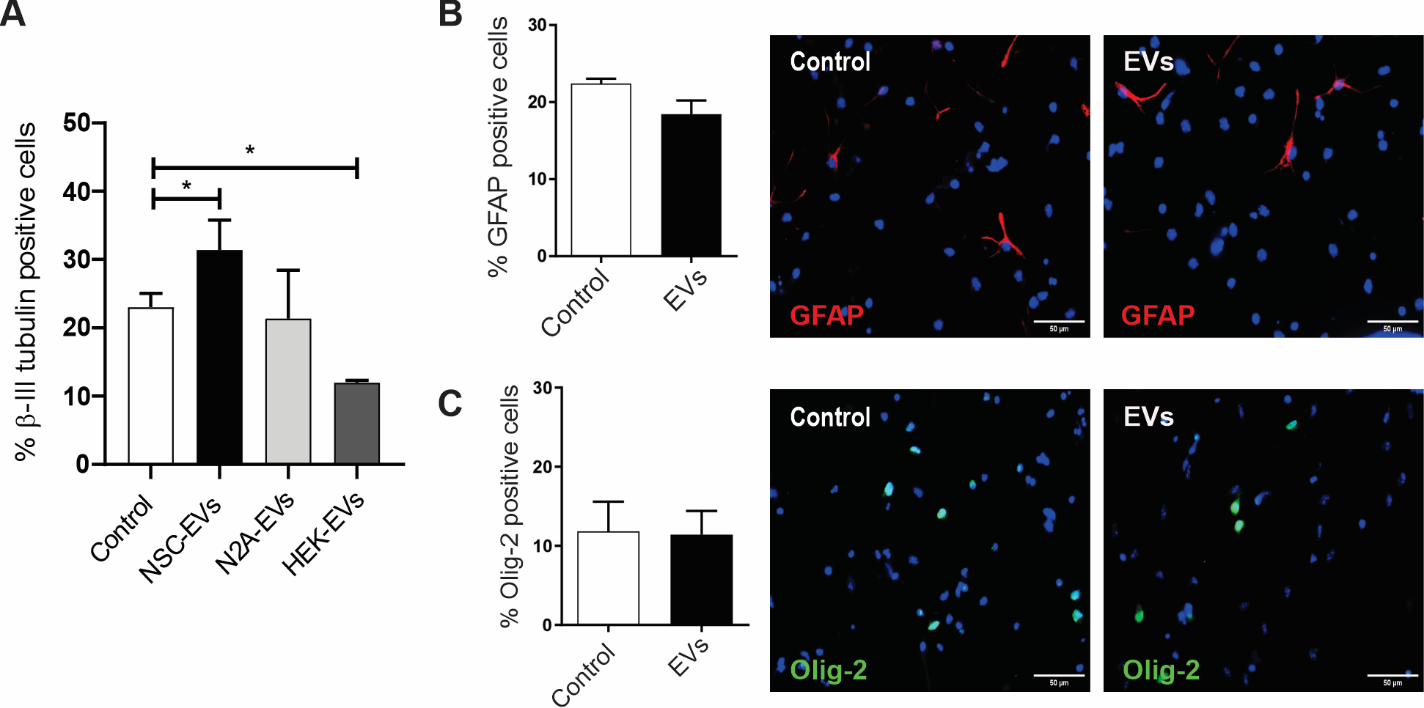


**Supplementary Figure 2: Effect of** **EVs in neuronal and glial differentiation.**  Neurosphere-derived cells were cultured during 72 hours under differentiation condition in presence or absence of EVs. **(A)** Percentage of βIII-tubulin positive cells analysed by immunocytochemistry coupled to fluorescence microscopy of in the presence of NSC-EVs, N2A-EVs or HEK-EVs **(B)** Quantification and representative images of the immunocytochemistry showing GFAP positive cells (red-astrocytes). **(C)** Quantification and representative images of the immunocytochemistry showing Olig-2 positive cells (green- oligodendrocytes). Data are presented as mean ± SEM of tree independent experiments. Scale bar: 50 µm. *p < 0.05


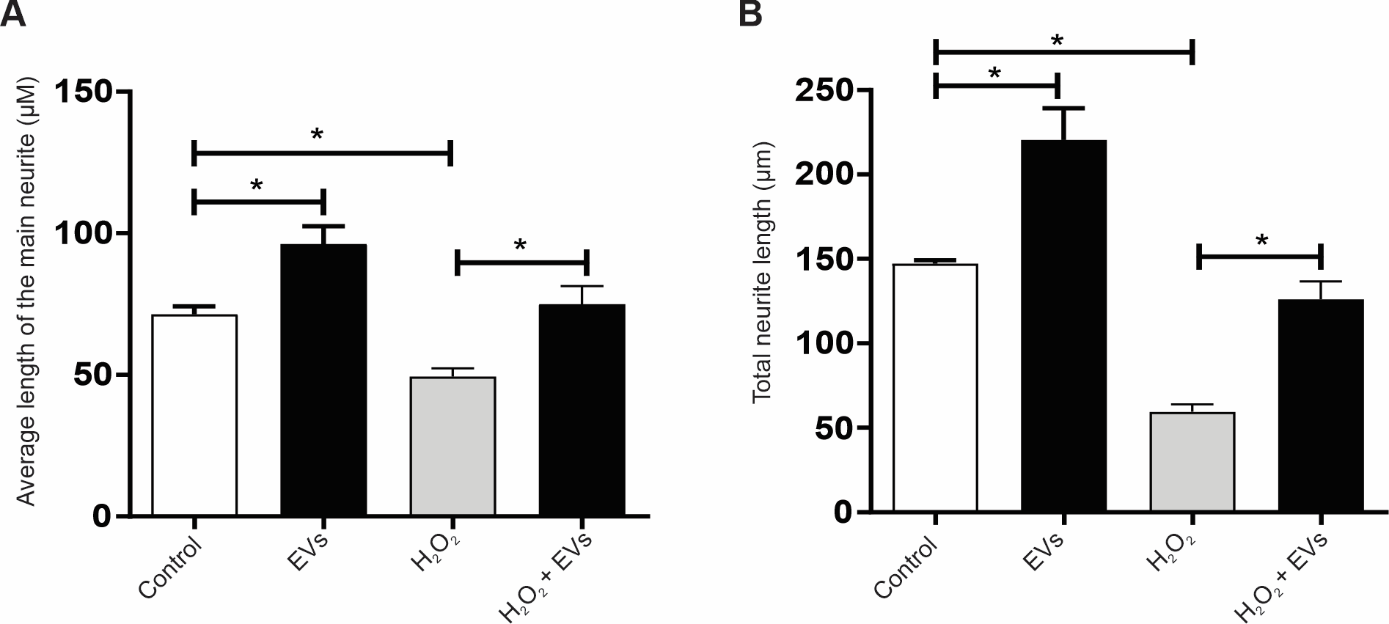


**Supplementary Figure 3:** **NSC-EVs promotes morphological parameters involved in neuronal development.** Neurosphere-derived cells cultured during 72 hours under differentiation condition in the presence or in the absence on NSC-EVs were immunostained with anti-βIII-tubulin antibody. Graphs represent the average length of the main neurite **(A)** and Total neurite length **(B)**. Data are presented as mean ± SEM of three independent experiments. *p < 0.05.

**Supplementary video**: EVs were labelled with Vybrant™ DiI Cell-Labelling Solution (Thermo Fisher) (1:9), the fate was evaluated by measuring red fluorescence with the confocal microscope (Zeiss LSM 880) and qualitative analyses were performed with Zen image acquisition software (Carl Zeiss). Same amount of the dye was dissolve in PBS in the absence of EVs and use as a control. **(Upper panel)** Representative images in brightfield microscopy of NSCs before labelling. **(Lower panel)** Video showing the increase in red fluorescence indicating the uptake of Dil-labeled EVs and the absence of fluorescence in the negative control condition.
